# Chronic Intermittent Hypobaric Hypoxia Improves Cardiac Function through Inhibition of Endoplasmic Reticulum Stress

**DOI:** 10.1038/s41598-017-08388-x

**Published:** 2017-08-11

**Authors:** Fang Yuan, Li Zhang, Yan-Qing Li, Xu Teng, Si-Yu Tian, Xiao-Ran Wang, Yi Zhang

**Affiliations:** 1 Department of Physiology, Hebei Medical University, Shijiazhuang, 050017 China; 2 Orthopedic Department of Third Hospital, Hebei Medical University, Shijiazhuang, 050000 China; 3 Department of Gynecology, Hebei Traditional Medicine Hospital, Shijiazhuang, 050011 China; 4 Hebei Key Lab of Laboratory Animal Science, Shijiazhuang, 050017 China; 5 Hebei Collaborative Innovation Center for Cardio-cerebrovascular Disease, Shijiazhuang, 050000 China

## Abstract

We investigated the role of endoplasmic reticulum stress (ERS) in chronic intermittent hypobaric hypoxia (CIHH)-induced cardiac protection. Adult male Sprague-Dawley rats were exposed to CIHH treatment simulating 5000 m altitude for 28 days, 6 hours per day. The heart was isolated and perfused with Langendorff apparatus and subjected to 30-min ischemia followed by 60-min reperfusion. Cardiac function, infarct size, and lactate dehydrogenase (LDH) activity were assessed. Expression of ERS molecular chaperones (GRP78, CHOP and caspase-12) was assayed by western blot analysis. CIHH treatment improved the recovery of left ventricular function and decreased cardiac infarct size and activity of LDH after I/R compared to control rats. Furthermore, CIHH treatment inhibited over-expression of ERS-related factors including GRP78, CHOP and caspase-12. CIHH-induced cardioprotection and inhibition of ERS were eliminated by application of dithiothreitol, an ERS inducer, and chelerythrine, a protein kinase C (PKC) inhibitor. In conclusion CIHH treatment exerts cardiac protection against I/R injury through inhibition of ERS via PKC signaling pathway.

## Introduction

Ischemic heart disease is one of the leading causes of morbidity and mortality for patients. Unless this trend is corrected, it will continue to be the mostly common cause of death among diseases in human being in 2030^1^. Although prompt reperfusion therapy in acute myocardial infarction enhances clinical outcome, residual morbidity and mortality are still relatively high^2, 3^. Furthermore, restoration of blood flow is associated with tissue injury. This reperfusion injury leads to lethal cell death and accounts for up to half of the ultimate infarct size^4, 5^. Up to date, an effective therapy that inhibits reperfusion injury has not yet been successfully implemented. Therefore, alternative therapeutic strategies have been intensively investigated to find the “cardio-protective” interventions for improving the survival ability of cardiac myocytes^6, 7^.

Chronic intermittent hypobaric hypoxia (CIHH) is a treatment with moderate hypoxia simulating high altitude interrupted by normoxia. Many studies have shown that CIHH has numerous beneficial effects, such as cardiac protection^8^, anti-hypertension^9^, anti-inflammation and modulation of immune function^10, 11^, and adjustment of metabolic dysfunctions^12, 13^. Our previous studies have demonstrated that CIHH promotes the recovery of cardiac function, diminishes infarction area, and antagonizes arrhythmia induced by ischemia/reperfusion (I/R)^8, 14^. Multiple mechanisms have been involved in the cardiac protection of CIHH, such as induction of heat-shock protein 70^15^, increase in coronary flow and myocardial capillary angiogenesis^16^, activation of adenosine triphosphate (ATP)-sensitive potassium channels, inhibition of mitochondrial permeability transition pores^17^, and maintenance of calcium homeostasis^18^. However, the precise mechanism underlying the cardiac protection of CIHH has not been fully elucidated.

Numerous pathological and environmental insults such as I/R can induce endoplasmic reticulum stress (ERS). During ERS the process of protein folding and posttranslational modification in the ER are influenced and may lead to build up mis-folded proteins in this organelle. Cells experiencing ERS must restore protein-folding capacity to match protein-folding demand quickly in order to survive^19, 20^. ERS is a double-edged sword which is protective at moderate level but harmful at severe level^21^. It has been shown that I/R injury in myocardium is markedly reduced by inhibition of ERS^22^ and by targeted deletion of p53-upregulated modulator of apoptosis (PUMA), an ERS response gene product^23^. Our previous study has shown that CIHH ameliorates ERS-mediated liver damage in a rat model of metabolic syndrome^24^. In this study, we test a hypothesis that CIHH protects heart against I/R injury through inhibiting ERS.

## Materials and Methods

### Chemicals

Chelerythrine, dithiothreitol (DTT) and 2,3,5-Triphenyltetrazolium chloride (TTC) were bought from Sigma Chemical Company (St Louis, MO). Antibodies against GRP78, CHOP, and caspase-12 were obtained from Abcam PLC (Cambridge, UK). Nitrocellulose membrane was obtained from Hybond-C (Amersham Life Science, UK) and the enhanced chemiluminescence (ECL) kit was provided by Beijing Applygen Technologies.

### Animal and CIHH treatment

Sixty adult male Sprague–Dawley rats (weight 300–350 g), obtained from the Animal Center in Hebei Medical University, were randomly divided into 2 groups: control group and CIHH treatment group (CIHH). The rats in control group were kept in the same environment as CIHH rats except hypoxia exposure. Body weight of rats was measured at a fixed time weekly. All rats were housed at room temperature with a natural light: dark cycle (12 h:12 h), and had free access to water and food.

All experiments were carried out in compliance with the Guide for the Care and Use of Laboratory Animals as adopted and promulgated by the U.S. National Institutes of Health, and was reviewed and approved by the Ethics Committee for the Use of Experimental Animals in Hebei Medical University.

### Langendorff perfusion and cardiac function recording

The rats were anesthetized with sodium pentobarbital (66 mg/kg, i.p.), and their hearts were quickly removed and retrogradely perfused through the aorta on a Langendorff apparatus with Krebs–Henseleit (K–H) buffer at constant pressure (76 mmHg) and temperature (37 °C). The ingredients of K-H buffer were (in mmol/L): NaCl 118, KCl 4.7, MgSO_4_ 1.2, CaCl_2_ 2.5, KH_2_PO_4_ 1.2, NaHCO_3_ 25 and glucose 11 (continuously bubbled with 95% O_2_ and 5% CO_2_, pH 7.4). A latex balloon-tipped catheter filled with saline was placed into the left ventricle through the left atrium and adjusted to a left ventricular end diastolic pressure (LVEDP) of 5–10 mmHg during the initial equilibration. The distal end of the catheter was connected to a pressure transducer (model Gould P23Db, AD Instrument Ltd., Australia) and the pressure signal was recorded with PowerLab system (AD Instrument). Left ventricular developed pressure (LVDP), LVEDP and the maximal differentials of LVDP (±LVdp/dt_max_) were continuously recorded. After 30 min stabilization with K-H buffer, the heart was subjected to 30 min no-flow ischemia, followed by 60 min reperfusion (or 120 min reperfusion for the determination of infarct size). The data were analyzed by using Chart software (AD Instrument).

### Determination of myocardial infarct size

Acute myocardial infarction was determined via 2,3, 5-Triphenyltetrazolium chloride (TTC) staining. Hearts were removed from Langendorff apparatus after I/R. Both atria and root of aorta were excised and ventricles were frozen at −20 °C for 1–2 h. Then hearts were cut into five transverse slices, incubated in phosphate buffer (pH 7.4) containing 1% TTC for 10 min at 37 °C for visualization. No detectable TTC staining was found in the infarct area of myocardium. The photos of slices were taken with a digital camera and analyzed by image processing system (Motic Med 6.0, MOTIC). The infarct size of myocardium was expressed as the percentage of the infarct size to the ventricular size.

### Assay of LDH

The severity of myocardial injury was reflected by the concentration of LDH in the coronary effluent. The effluent was collected before ischemia and 1st, 5th, 10th, 20th, 30th, 40th, 50th and 60th min of reperfusion in all rats, respectively, and the concentration of LDH was assayed using a commercial LDH kit (Nanjing Jiancheng Bioengineering Institute, China).

### Western blot analysis

Samples were homogenized in ice-cold lysis buffer. Total proteins were extracted from the hearts, and equal amounts of protein (100 μg/lane) were loaded, subjected to electrophoresis on SDS-polyacrylamide gel and transferred onto nitrocellulose membrane. Membranes were blocked with nonfat milk and incubated with primary antibodies anti-GRP78 (1:10,000), anti-CHOP (1:1,000), anti-caspase-12 (1:500) and anti-GADPH (1:2000) at 4 °C overnight. Then the membranes were incubated with secondary antibody for 1 h at room temperature. Blots were developed by the chemiluminescent detection method (Amersham ECL). The films were scanned and analyzed by NIH image software. The protein contents were normalized to that of GADPH. All experiments were repeated three times.

### Statistical Analysis

All data were expressed as mean ± SD. Significant differences were determined by one-way ANOVA followed by a Dunnett’s test, or two-way ANOVA followed by a Bonferroni post hoc test. A Student t-test was used when only two groups were compared. *P* < 0.05 was considered statistically significant. Sample size (n) represented the number of independent experiments.

## Results

### Effect of CIHH on heart weight

The body weight of rats increased during the period of the experiment and there was no significant difference in body weight between control and CIHH rats (*P* > 0.05, Table 1). The ratios of ventricle weight to whole body weight (VW/BW), left ventricle weight to whole body weight (LV/BW), right ventricle weight to whole body weight (RV/BW), and right ventricle weight to left ventricle weight (RV/LV) in CIHH rats were unchanged significantly compared with those in control rats (*P* > 0.05, Table 2).

**Table 1:** Effect of CIHH on body weight in rats.

Treating days	0 day (g)	7 day (g)	14 day (g)	21 day (g)	28 day (g)
Control	135.7 ± 7.1	170.3 ± 17.2	224.3 ± 17.5	262.9 ± 22.2	326.9 ± 28.0
CIHH	135.8 ± 5.9	160.9 ± 12.4	209.8 ± 14.5	246.0 ± 18.8	321.1 ± 21.2

All data expressed as mean ± SD, *n* = 6 for each group.

**Table 2:** Effect of CIHH on heart weight in rats.

Group	VW/BW (%)	RV/BW (%)	LV/BW (%)	RV/LV (%)
Control	0.36 ± 0.04	0.07 ± 0.01	0.27 ± 0.03	25.17 ± 2.85
CIHH	0.39 ± 0.04	0.08 ± 0.01	0.28 ± 0.02	27.03 ± 3.71

BW: body weight; VW: ventricles weight; RV: right ventricle weight; LV: left ventricle weight. All Data were expressed as mean ± SD, *n* = 6 for each group.

### Cardiac protection of CIHH during I/R

**Figure 1:**
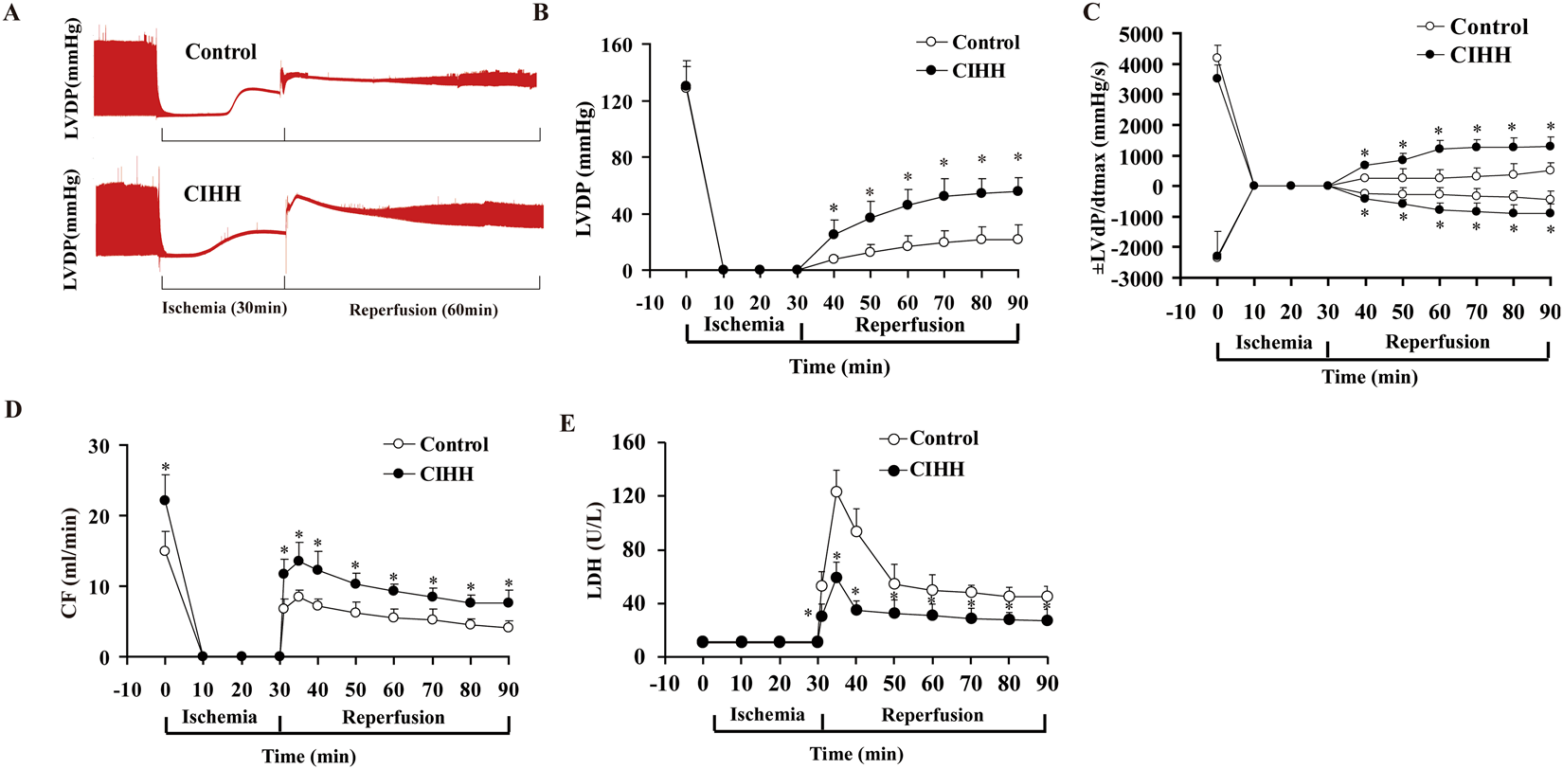
Effect of CIHH on the cardiac function, Coronary flow, and LDH in isolated rat hearts. CIHH treatment consisted of a procedure of 30 min ischemia and 60 min reperfusion. (**A**) Original recordings of left ventricular pressure. (**B**,**C**) Effect of CIHH on LVDP and ± LVdP/dt_max_. (**D**) Effect of CIHH on coronary flow. (**E**) Effect of CIHH on LDH activity in coronary effluent. CIHH: chronic intermittent hypobaric hypoxia. All data were expressed as mean ± SD, *n* = 6 for each group. *P < 0.05 *vs* control group.

CIHH did not significantly change the basal parameters of left ventricular function, but effectively promoted the left ventricular function recovery from I/R. The recovery of LVDP, +LVdp/dt_max_ and −LVdp/dt_max_ in CIHH rats was 42.8, 38.1 and 34.6% at 60 min, higher than 19.0, 18.2 and 17.4% in control rats, respectively (*P* < 0.05, Fig. 1 A,B and C). In addition, the coronary flow in CIHH rats was significantly higher than that in control rats under both basal and reperfusion conditions (*P* < 0.05, Fig. 1D).

**Figure 2:**
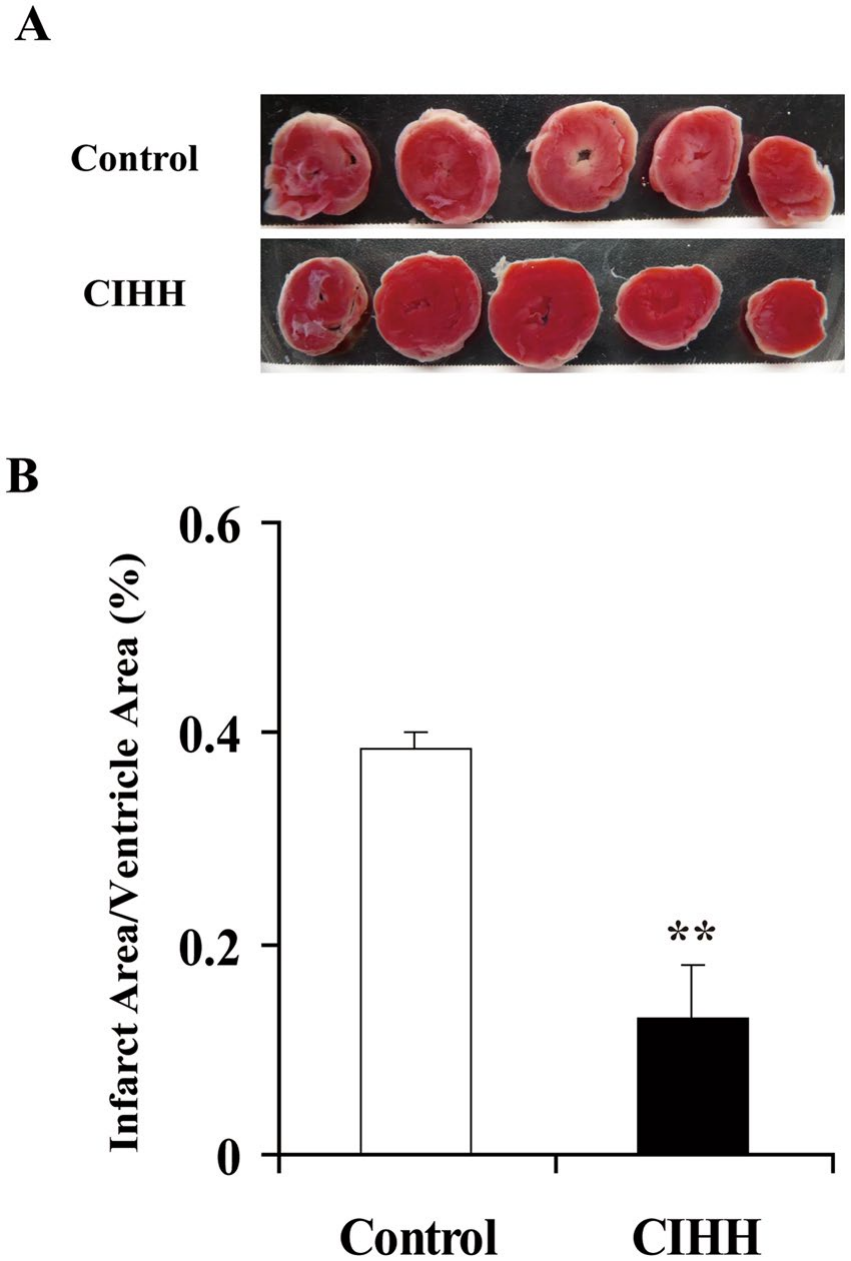
Effect of CIHH on the infarct size in isolated rat hearts. (**A**) Representative 2,3,5-Triphenyltetrazolium chloride (TTC) staining of heart slices after I/R. (**B**) Effect of CIHH on infarct size. All data were expressed as mean ± SD, *n* = 4 for each group. **P < 0.01 *vs* control group.

After 30-min ischemia and 120-min reperfusion, myocardial infarct area was 12.8 ± 2% in CIHH rats and 38.4 ± 5% in control rats (*P* < 0.05, Fig. 2). We also measured LDH concentration in the coronary effluent to reflect the severity of myocardial injury. The LDH concentration in coronary effluent in CIHH rats was significantly lower than that in control rats (*P *< 0.05, Fig. 1E). These results indicate that CIHH diminishes infarct area and LDH activity.

### Role of ERS in CIHH cardioprotection

**Figure 3:**
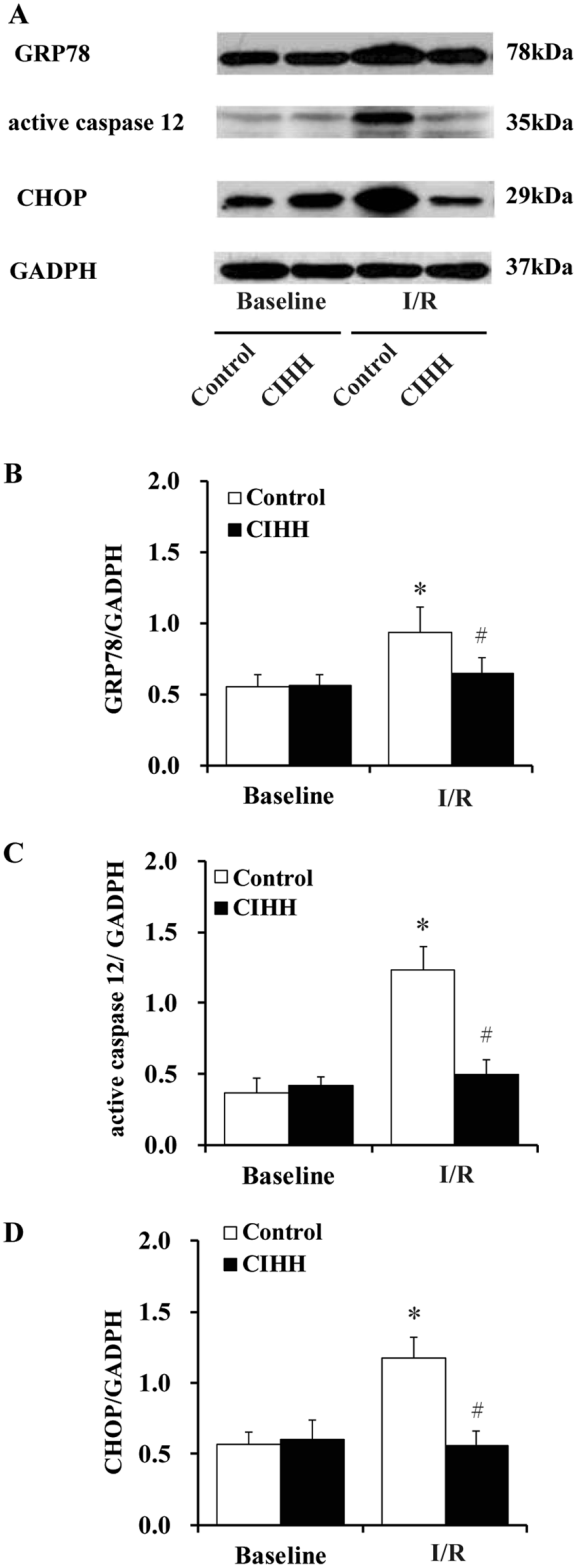
CIHH inhibited the increase of ERS marker proteins induced by I/R. (**A**) Representative protein expression of GRP78, active caspase-12, CCAAT/enhancer binding protein homologous protein (CHOP); (**B**–**D**) Quantitative analysis of protein expression of GRP78, active caspase 12, and CHOP. All data were expressed as mean ± SD, *n* = 6 for each group. *P < 0.05 *vs* each corresponding baseline group. ^#^ *P* < 0.05 *vs* CIHH I/R group.

**Figure 4:**
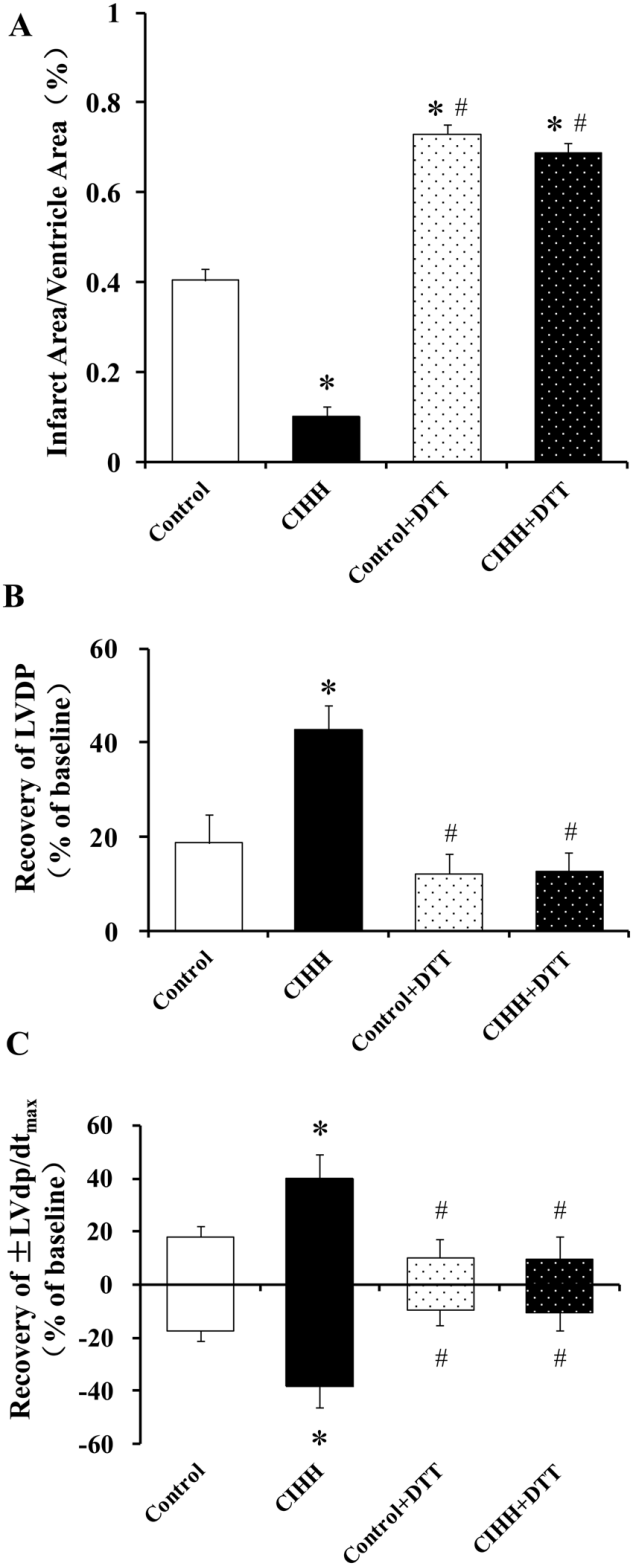
DTT, an ERS inducer blocked CIHH-induced cardioprotection in isolated rat hearts subjected to 30 min of ischemia and 60 min of reperfusion. (**A**) Infarct size. (**B**) LVDP. (**C**) ±LVdp/dt_max_. All data were expressed as mean ± SD, *n* = 6 for each group. *P < 0.05 *vs* control group. ^#^ *P* < 0.05 CIHH group.

**Figure 5:**
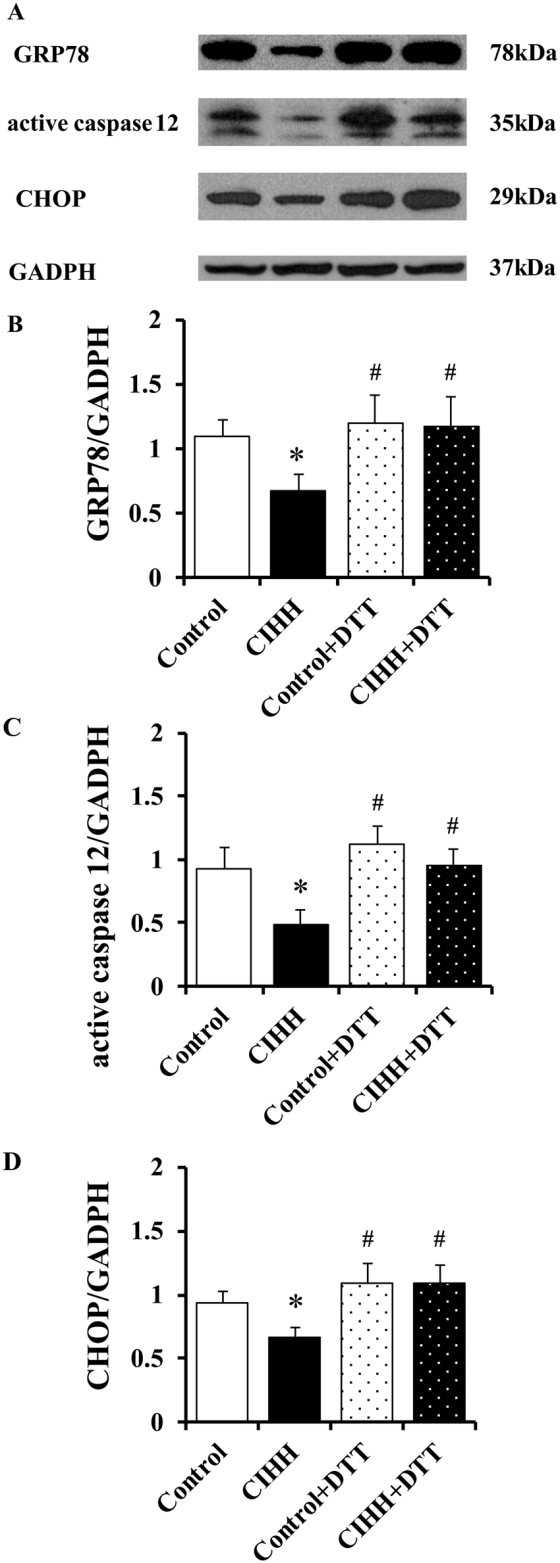
Effect of DTT on expression of ERS marker proteins. (**A**) Original gel images showing protein expression of GRP78, active caspase-12, CHOP; (**B**–**D**) Quantitative analysis of protein expression of GRP78, active caspase 12, and CHOP. All data were expressed as mean ± SD, *n* = 6 for each group. *P < 0.05 *vs* control group. ^#^ *P* < 0.05 *vs* CIHH group.

We then determined ERS levels by measuring ERS marker proteins GRP78, activated caspase-12, and CHOP. The expression levels GRP78, activated caspase-12 and CHOP did not differ between CIHH and control rats under basic condition (*P* > 0.05, Fig. 3). The myocardial protein expression levels of GRP78, activated caspase-12, and CHOP were significantly enhanced after I/R. However, the increase of these ERS marker proteins was alleviated in CIHH rats compared with control rats (*P* < 0.05, Fig. 3). The cardiac protection of CIHH was blocked by pretreatment with DTT, an ERS inducer, before ischemia. Inversely pretreatment with DTT induced tissue damage in control rats (*P *< 0.05, Figs 4 and 5).

### Role of PKC in CIHH-induced cardiac protection

**Figure 6:**
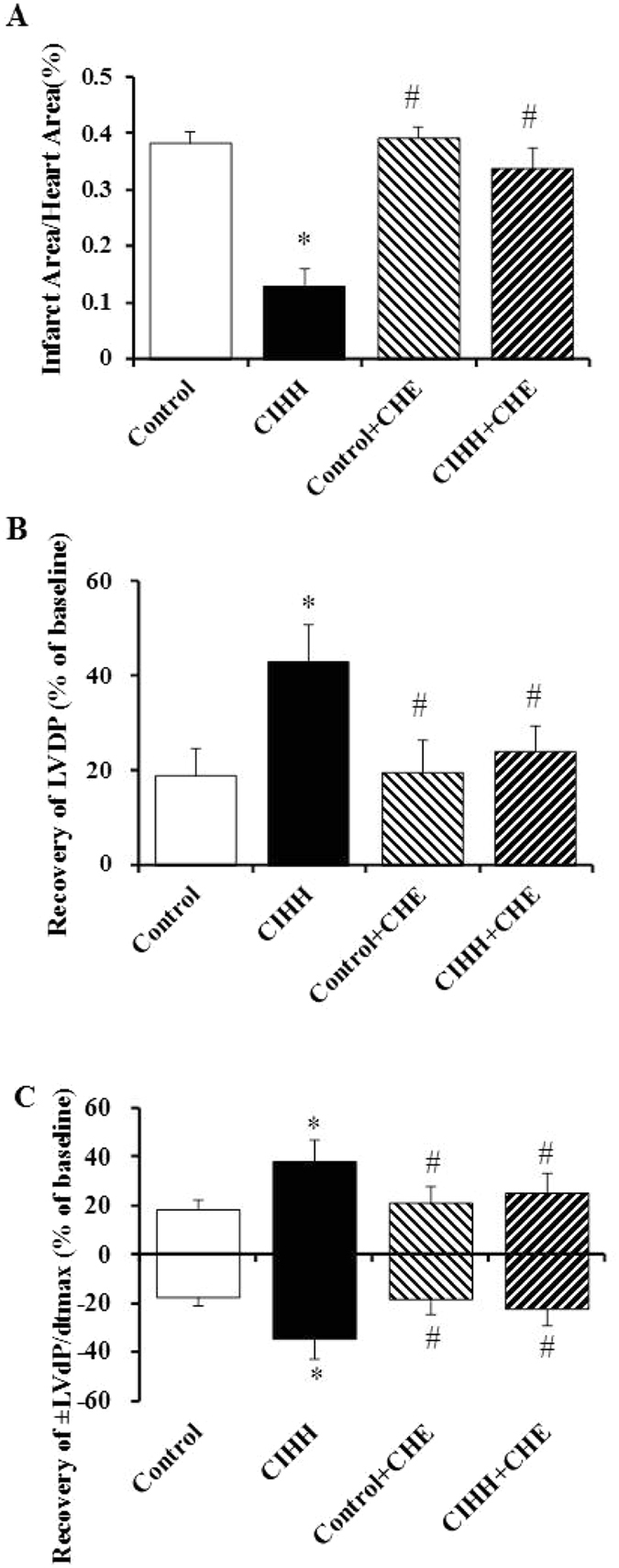
Inhibtion of PKC blocked the CIHH-induced cardiac protection in isolated rat hearts subjected to 30-min ischemia and 60-min reperfusion. (**A**) Infarct size. (**B**) LVDP. (**C**) ± LVdp/dt_max_. All data were expressed as mean ± SD, *n* = 6 for each group. *P < 0.05 *vs* control group. ^#^ *P* < 0.05 *vs* CIHH group.

**Figure 7:**
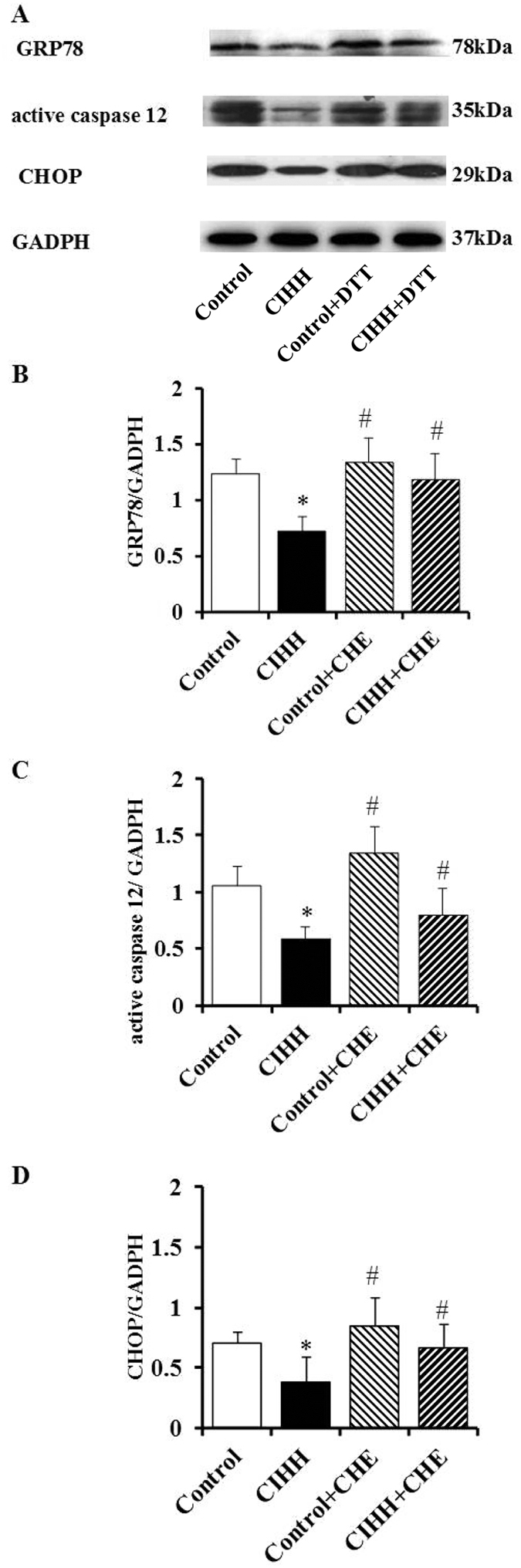
Effect of PKC inhibitor on expression of ERS marker proteins. (**A**) Representative protein expression of GRP78, active caspase-12, CCAAT/enhancer binding protein homologous protein (CHOP); (**B**–**D**) Quantitative analysis of protein expression of GRP78, active caspase 12, and CHOP. All data were expressed as mean ± SD, *n* = 6 for each group. *P < 0.05 *vs* control group. ^#^ *P* < 0.05 *vs* CIHH group.

We determined the role of protein kinase C (PKC) in the cardiac protection and reduction of GRP78, active caspase-12 and CHOP in CIHH rats. CIHH-induced cardiac protection and reduction of GRP78, active-caspase12 and CHOP were blocked by chelerythrine (5 × 10^−6^ mol/L), a PKC inhibitor (*P* < 0.05, Figs 6 and 7).

## Discussion

In this study, we found that CIHH effectively protected the heart against I/R injury by promoting the recovery of left ventricular function, diminishing infarct area, and decreasing LDH concentration. In addition, CIHH treatment inhibited I/R-induced ERS as indicated by a downregulation of ERS marker proteins. Such an effect was reversed by activation of ERS or inhibition of PKC signaling.

Previous studies have demonstrated that ERS is involved in the pathogenesis of various cardiovascular diseases including myocardium I/R injury^20^. Inhibition of ERS contributes to cardiovascular protection. In this regard, inhibition of ERS with intermedin or ghrelin protects the myocardium against I/R injury^22, 25^. We found that I/R resulted in ERS and CIHH inhibited ERS to exert cardiac protection. However ERS inducers aggravated the cardiac I/R damage in control rats and reversed the cardiac protective effect in CIHH-treated rats. These results indicate that CIHH ameliorates myocardium I/R injury though inhibiting myocardial ERS.

It is known that different perturbations, such as ischemia or hypoxia, at the cellular level disrupt ER homeostasis and produce an accumulation of unfolded proteins in the lumen of ER^26^. ERS can be alleviated by the unfolded protein response (UPR), a series of adaptive mechanisms, through promoting capability of protein folding and clearance to reduce misfolded proteins^27^. In prolonged and irreversible ERS, however, cells that become irreversibly damaged are eliminated by apoptosis^28^. Thus, apoptosis is an important factor in I/R-induced cardiac damage. A number of studies have shown that inhibition of apoptosis contributes to cardiac protection against I/R injury^29–31^. CIHH treatment protects the heart against I/R injury through attenuating I/R-induced apoptosis due to increasing the ratio of Bcl-2/Bax proteins^32^. Consistent with above-mentioned reports, the present study showed that CIHH downregulated expression of CHOP and active-caspase12, two key components of apoptosis pathway, in favor of a cardioprotective role of CIHH.

PKC signaling pathway plays a critical role in regulation of ERS^33^. Through interacting with calcium-sensing receptors, PKC protects post-conditioned cardiomyocytes from programmed cell death by inhibiting ERS^32, 33^. Our findings demonstrated that inhibition of PKC impaired the protective effect of CIHH against myocardial I/R injury. It has been shown that the effect of different PKC isoforms on ERS was variable. For example, Dong *et al*. reported that the inhibitory effect of PKC on myocardial ERS was mainly mediated by PKCε^33^. Madaro *et al*. found that PKC_θ_, as an ERS sensor in skeletal muscle, was required for ERS-dependent autophagy activation^34^. Larroque-Cardoso *et al*. revealed that PKC_δ_ acted as a key regulator of oxidized low-density lipoproteins-induced ERS-mediated apoptosis in human vascular smooth muscle cells^35^. Therefore, future studies are needed to determine the PKC isoforms involved in the CIHH-induced cardiac protection.

## Conclusion

In conclusion, this study demonstrated for the first time that CIHH protects hearts against ischemia/reperfusion injury through inhibiting myocardial ERS via PKC signaling pathways. This finding may help develop new strategies of cardiac protection against ischemic cardiac injuries.
